# Supplementary material for: Transfer of a Multiclass Method for over 60 Antibiotics in Food from High Resolution to Low Resolution Mass Spectrometry
Source: Molecules. 2019 Aug 13;24(16):2935. doi: 10.3390/molecules24162935 (PMC6720601; doi:10.3390/molecules24162935)
Supplement: Supplementary file 1 [file molecules-24-02935-s001.pdf]

## Supplementary Material

# TRANSFER OF A MULTICLASS METHOD FOR OVER 60 ANTIBIOTICS IN FOOD FROM HIGH RESOLUTION TO LOW RESOLUTION MASS SPECTROMETRY

Danilo Giusepponi[1], Fabiola Paoletti[1], Carolina Barola[1], Simone Moretti[1], Giorgio Saluti[1], Federica Ianni[2], Roccaldo Sardella[2], Roberta Galarini[1]

[1]Istituto Zooprofilattico Sperimentale dell'Umbria e delle Marche "Togo Rosati", 06126 Perugia, Italy
[2]University of Perugia, Department of Pharmaceutical Sciences, 06123 Perugia, Italy

**Table S1- Validation plan in bovine muscle**

| Analyte spiking level (µg $kg^{-1}$) | Concentration of analyte solution (µg $mL^{-1}$) | Added volume of analyte solution (µL) | IS spiking level (µg $kg^{-1}$) | Concentration of IS solution (µg $mL^{-1}$) | Added volume of IS solution (µL) | Number of fortified samples/day | Final volume (mL) | Dilution |
|---|---|---|---|---|---|---|---|---|
| 10 | 1 | 15 | 10 | 1 | 15 | 4 | 1.5 | 1 |
| 33 | 1 | 50 | 10 | 1 | 15 | 4 | 1.5 | 1 |
| 100 | 1 | 150 | 10 | 1 | 15 | 4 | 1.5 | 1 |
| 150 | 1 | 225 | 10 | 1 | 15 | 4 | 1.5 | 1 |
| 333[a] | 10 | 50 | 100 | 10 | 15 | 4 | 15 | 10 |
| 1000[a] | 10 | 150 | 100 | 10 | 15 | 4 | 15 | 10 |
| 1500[a] | 10 | 225 | 100 | 10 | 15 | 4 | 15 | 10 |

[a] Starting from 333 µg $kg^{-1}$, for practical reasons (added volumes), only the analytes with MRL > 100 µg $kg^{-1}$ have been added and the final extract diluted 1:10 prior to the injection in the LC system

**Table S2- Validation plan in milk**

| Analyte spiking level (µg kg$^{-1}$) | Concentration of analyte solution (µg mL$^{-1}$) | Added volume of analyte solution (µL) | IS spiking level (µg kg$^{-1}$) | Concentration of IS solution (µg mL$^{-1}$) | Added volume of IS solution (µL) | Number of fortified samples/day | Final volume (mL) | Dilution |
|---|---|---|---|---|---|---|---|---|
| 2[a] | 0.1 | 30 | 10 | 1 | 15 | 4 | 1.5 | 1 |
| 10 | 1 | 15 | 10 | 1 | 15 | 4 | 1.5 | 1 |
| 33 | 1 | 50 | 10 | 1 | 15 | 4 | 1.5 | 1 |
| 100 | 1 | 150 | 10 | 1 | 15 | 4 | 1.5 | 1 |
| 150 | 1 | 225 | 10 | 1 | 15 | 4 | 1.5 | 1 |
| 333[b] | 10 | 50 | 100 | 10 | 15 | 4 | 15 | 10 |

[a] This concentration was done only for amoxicillin, ampicillin and penicillin G; [b] For practical reasons (added volumes), only the analytes with MRL > 100 µg kg$^{-1}$ have been added and the final extract diluted 1:10 prior to the injection in the LC system

**Table S3- Linearity studies in matrix (matrix-matched curves)**

| Antibiotic class | Analyte | Linearity range: muscle ($\mu g\ kg^{-1}$) | Linearity range: milk ($\mu g\ kg^{-1}$) |
|---|---|---|---|
| Amphenicols | Florfenicolamine | 10-150 | 2-150 |
| | Thiamphenicol | 10-150 | 2-150 |
| | Florfenicol | 33-150 | 10-150 |
| Beta lactams | Amoxicillin | 2-150 | 2-150 |
| | Ampicillin | 2-150 | 2-150 |
| | Cefacetrile | 33-150 | 100-150 |
| | Cefalexin | 2-150 | 2-150 |
| | Cefalonium | 2-150 | 10-150 |
| | Cephapirin | 2-150 | 2-150 |
| | Cefazolin | 2-150 | 10-150 |
| | Cefoperazone | 2-150 | 2-150 |
| | Cefquinome | 2-150 | 10-150 |
| | Ceftiofur | 2-150 | 2-150 |
| | Cloxacillin | 2-150 | 2-150 |
| | Desacetylcephapirin | 2-150 | 2-150 |
| | Dicloxacillin | 2-150 | 10-150 |
| | Nafcillin | 2-150 | 2-150 |
| | Oxacillin | 2-150 | 10-150 |
| | Penicillin G | 2-150 | 10-150 |
| | Penicillin V | 2-150 | 2-150 |
| Quinolones | Ciprofloxacin | 10-150 | 10-150 |
| | Danofloxacin | 10-150 | 10-150 |
| | Difloxacin | 10-150 | 10-150 |
| | Enrofloxacin | 10-150 | 10-150 |
| | Flumequine | 10-150 | 10-150 |
| | Marbofloxacin | 10-150 | 10-150 |
| | Nalidixic acid | Not evaluated | 10-150 |
| | Norfloxacin | Not evaluated | 10-150 |
| | Oxolinic acid | 10-150 | 10-150 |

| | | | |
|---|---|---|---|
| | Sarafloxacin | 10-150 | 10-150 |
| Macrolides | 3-O-Acetyltylosin | 2-150 | 2-150 |
| | Erythromycin | 10-150 | 2-150 |
| | Gamithromycin | 2-150 | 10-150 |
| | Neospiramycin | 2-150 | 2-150 |
| | Spiramycin | 10-150 | 2-150 |
| | Tylosin A | 10-150 | 2-150 |
| | Tildipirosin | 2-150 | 10-100 |
| | Tilmicosin | 10-150 | 10-150 |
| | Tylvalosin | 10-150 | 2-150 |
| | Tulathromycin marker | 2-150 | 10-100 |
| | Tulathromycin | 10-150 | 10-150 |
| Sulfonamides | Sulfadiazine | 2-150 | 2-150 |
| | Sulfadimethoxine | 2-150 | 2-150 |
| | Sulfaguanidine | 33-150 | 2-150 |
| | Sulfamerazine | 10-150 | 2-150 |
| | Sulfamethazine | 2-150 | 2-150 |
| | Sulfamethoxazole | 2-150 | 10-150 |
| | Sulfamonomethoxine | 2-150 | 2-150 |
| | Sulfanilamide | 33-100 | 2-150 |
| | Sulfapyridine | 2-150 | 2-150 |
| | Sulfaquinoxaline | 10-150 | 2-150 |
| | Sulfathiazole | 2-150 | 2-150 |
| Diamino pymidines | Trimethoprim | 2-150 | 10-150 |
| Tetracyclines | 4-epi-chlortetracycline | 10-150 | 2-150 |
| | 4-epi-oxytetracycline | 2-150 | 2-150 |
| | 4-epi-tetracycline | 2-150 | 2-150 |
| | Doxycycline | 2-150 | 2-150 |
| | Chlortetracycline | 10-150 | 2-150 |
| | Oxytetracycline | 2-150 | 2-150 |
| | Tetracycline | 10-150 | 10-150 |
| Lincosamides | Lincomycin | 2-150 | 10-150 |

| | | | |
|---|---|---|---|
| Rifampicins | Rifaximin | 10-150 | 2-150 |
| Pleuromutilins | Tiamulin | 2-150 | 2-150 |
| | Valnemulin | 10-150 | 2-150 |

## Table S4- Calibration data (matrix-matched curves) of six representative analytes

| Concentration (µg kg$^{-1}$) | Muscle | | | Milk | | |
|---|---|---|---|---|---|---|
| **Danofloxacin** | **Mean peak area[a]** | **Back-calculated concentration[b] (µg kg$^{-1}$)** | **Deviation (%)[c]** | **Mean peak area[a]** | **Back-calculated concentration[b] (µg kg$^{-1}$)** | **Deviation (%)[c]** |
| 2 | - | - | - | - | - | - |
| 10 | 2207097 | 12 | 20 | 1732977 | 11 | 12 |
| 33 | 6301830 | 34 | 4.1 | 5532588 | 36 | 7 |
| 100 | 19501848 | 106 | 6.3 | 16212978 | 105 | 5 |
| 150 | 27271102 | 149 | -0.9 | 23084541 | 149 | -1 |
| $R^2$ | 0.998 | | | 0.9988 | | |
| Slope | 183505 | | | 154760 | | |
| intercept | 302955 | | | 234367 | | |
| **Doxycycline** | **Mean peak area[a]** | **Back-calculated concentration[b] (µg kg$^{-1}$)** | **Deviation (%)[c]** | **Mean peak area[a]** | **Back-calculated concentration[b] (µg kg$^{-1}$)** | **Deviation (%)[c]** |
| 2 | 113808 | 1.7 | -15 | 64792 | 1.8 | -8.9 |
| 10 | 613831 | 9.1 | -8.7 | 305648 | 8.6 | -14 |
| 33 | 2064390 | 31 | -6.9 | 1086503 | 31 | -7.4 |
| 100 | 6827497 | 102 | 1.6 | 3431807 | 97 | -3.5 |
| 150 | 9970481 | 148 | -1.1 | 5361000 | 151 | 0.5 |
| $R^2$ | 0.9995 | | | 0.9993 | | |
| Slope | 67208 | | | 35564 | | |
| intercept | -39377 | | | -40255 | | |
| **Oxacillin** | **Mean peak area[a]** | **Back-calculated concentration[b] (µg kg$^{-1}$)** | **Deviation (%)[c]** | **Mean peak area[a]** | **Back-calculated concentration[b] (µg kg$^{-1}$)** | **Deviation (%)[c]** |
| 2 | 134310 | 1.9 | -2.7 | - | - | - |
| 10 | 689885 | 10 | 0 | 70005 | 10 | 4 |
| 33 | 2384304 | 35 | 4.7 | 247580 | 37 | 12 |
| 100 | 6900456 | 100 | 0 | 691866 | 103 | 3 |
| 150 | 10364762 | 150 | 0.1 | 1006262 | 150 | 0 |
| $R^2$ | 0.9999 | | | 0.9991 | | |
| Slope | 68998 | | | 6712 | | |
| intercept | 19900 | | | 9825 | | |

| Spiramycin | Mean peak area[a] | Back-calculated concentration[b] (µg kg$^{-1}$) | Deviation (%)[c] | Mean peak area[a] | Back-calculated concentration[b] (µg kg$^{-1}$) | Deviation (%)[c] |
|---|---|---|---|---|---|---|
| 2 | - | - | - | 52546 | 2.4 | 20 |
| 10 | 533578 | 10 | 2.4 | 211964 | 9.7 | -3.3 |
| 33 | 1620556 | 31 | -5.8 | 788059 | 36 | 9.0 |
| 100 | 5355690 | 103 | 2.8 | 2343962 | 107 | 7.0 |
| 150 | 7728987 | 148 | -1.1 | 3222096 | 147 | -2.0 |
| $R^2$ | 0.9991 | | | 0.9970 | | |
| Slope | 52105 | | | 21915 | | |
| intercept | -5605 | | | 25611 | | |
| **Sulfamethazine** | **Mean peak area[a]** | **Back-calculated concentration[b] (µg kg$^{-1}$)** | **Deviation (%)[c]** | **Mean peak area[a]** | **Back-calculated concentration[b] (µg kg$^{-1}$)** | **Deviation (%)[c]** |
| 2 | 201038 | 2.0 | 1.5 | 74659 | 1.9 | -6.0 |
| 10 | 1132098 | 11 | 14 | 421374 | 11 | 6.1 |
| 33 | 3525227 | 36 | 7.9 | 1493884 | 38 | 14 |
| 100 | 10728282 | 108 | 8.3 | 4114759 | 104 | 3.6 |
| 150 | 14534896 | 147 | -2.1 | 5921403 | 149 | -0.6 |
| $R^2$ | 0.9961 | | | 0.9987 | | |
| Slope | 99023 | | | 39714 | | |
| intercept | 151609 | | | 51741 | | |
| **Tiamulin** | **Mean peak area[a]** | **Back-calculated concentration[b] (µg kg$^{-1}$)** | **Deviation (%)[c]** | **Mean peak area[a]** | **Back-calculated concentration[b] (µg kg$^{-1}$)** | **Deviation (%)[c]** |
| 2 | 1266776 | 1.7 | -15 | 843106 | 2.1 | 7.0 |
| 10 | 8166183 | 11 | 9.5 | 3941489 | 10 | 0.1 |
| 33 | 24718486 | 33 | 0.5 | 13038241 | 33 | 0.3 |
| 100 | 78910016 | 106 | 5.8 | 39452787 | 100 | 0.2 |
| 150 | 109836949 | 147 | -1.8 | 59075828 | 150 | 0 |
| $R^2$ | 0.9979 | | | 1.0000 | | |
| Slope | 745604 | | | 393844 | | |
| intercept | 490855 | | | 27922 | | |

[a] Each matrix-matched point was injected three times; [b]Measured (calculated) concentration using the calibration curve ($C_{measured}$); [c]Deviation (%) of the back-calculated concentration from the true one: $C_{measured}$-$C_{true}$) x 100/$C_{true}$. In the relevant region the deviation should not be more than ±20% [32]

**Table S5 – MRL (µg kg$^{-1}$), decision limits (µg kg$^{-1}$) and detection capabilities (µg kg$^{-1}$) of the 64 tested antibiotics in the main food-producing species**

| | Analyte | Bovine | | | Porcine | | | poultry | | | Ovi-caprine | | |
|---|---|---|---|---|---|---|---|---|---|---|---|---|---|
| | | MRL | CCα | CCβ | MRL | CCα | CCβ | MRL | CCα | CCβ | MRL | CCα | CCβ |
| Amphenicols | Florfenicolamine | 200 | 220 | 242 | 300 | 330 | 363 | 100 | 110 | 121 | 200 | 220 | 242 |
| | Florfenicol | 200 | 252 | 319 | 300 | 379 | 478 | 100 | 126 | 159 | 200 | 252 | 319 |
| | Thiamphenicol | 50 | 59 | 70 | 50 | 59 | 70 | 50 | 59 | 70 | 50 | 59 | 70 |
| Beta lactams | Amoxicillin | 50 | 55 | 61 | 50 | 55 | 61 | 50 | 55 | 61 | 50 | 55 | 61 |
| | Ampicillin | 50 | 57 | 64 | 50 | 57 | 64 | 50 | 57 | 64 | 50 | 57 | 64 |
| | Cefacetrile | - | 33 | 33 | - | 33 | 33 | - | 33 | 33 | - | 33 | 33 |
| | Cefalexin | 200 | 228 | 259 | - | 10 | 10 | - | 10 | 10 | - | 10 | 10 |
| | Cefalonium | - | 10 | 10 | - | 10 | 10 | - | 10 | 10 | - | 10 | 10 |
| | Cephapirin | 50 | 56 | 62 | - | 10 | 10 | - | 10 | 10 | - | 10 | 10 |
| | Cefazolin | - | 10 | 10 | - | 10 | 10 | - | 10 | 10 | - | 10 | 10 |
| | Cefoperazone | - | 10 | 10 | - | 10 | 10 | - | 10 | 10 | - | 10 | 10 |
| | Cefquinome | 50 | 56 | 63 | 50 | 56 | 63 | - | 10 | 10 | - | 10 | 10 |
| | Ceftiofur | 1000 | 1159 | 1343 | 1000 | 1159 | 1343 | - | 10 | 10 | 1000 | 1159 | 1343 |
| | Cloxacillin | 300 | 354 | 418 | 300 | 354 | 418 | 300 | 354 | 418 | 300 | 354 | 418 |
| | Desacetylcephapirin | 50 | 56 | 63 | - | 10 | 10 | - | 10 | 10 | - | 10 | 10 |
| | Dicloxacillin | 300 | 349 | 406 | 300 | 349 | 406 | 300 | 349 | 406 | 300 | 349 | 406 |
| | Nafcillin | 300 | 340 | 386 | - | 10 | 10 | - | 10 | 10 | 300 | 340 | 386 |
| | Oxacillin | 300 | 354 | 418 | 300 | 354 | 418 | 300 | 354 | 418 | 300 | 354 | 418 |
| | Penicillin G | 50 | 57 | 66 | 50 | 57 | 66 | 50 | 57 | 66 | 50 | 57 | 66 |
| | Penicillin V | - | 10 | 10 | 25 | 30 | 35 | 25 | 30 | 35 | - | 10 | 10 |
| Quinolones | Oxolinic Acid | 100 | 111 | 123 | 100 | 111 | 123 | 100 | 111 | 123 | 100 | 111 | 123 |
| | Ciprofloxacin | 100 | 114 | 129 | 100 | 114 | 129 | 100 | 114 | 129 | 100 | 114 | 129 |
| | Danofloxacin | 200 | 224 | 250 | 100 | 112 | 125 | 200 | 224 | 250 | 200 | 224 | 250 |
| | Difloxacin | 400 | 449 | 503 | 400 | 449 | 503 | 300 | 336 | 377 | 400 | 449 | 503 |
| | Enrofloxacin | 100 | 111 | 123 | 100 | 111 | 123 | 100 | 111 | 123 | 100 | 111 | 123 |
| | Flumequine | 200 | 224 | 251 | 200 | 224 | 251 | 400 | 448 | 502 | 200 | 224 | 251 |

| | | | | | | | | | | | | | |
|---|---|---|---|---|---|---|---|---|---|---|---|---|---|
| Quinolones | Marbofloxacin | 150 | 167 | 186 | 150 | 167 | 186 | - | 10 | 10 | - | 10 | 10 |
| | Sarafloxacin | - | 10 | 10 | - | 10 | 10 | - | 10 | 10 | - | 10 | 10 |
| | Nalidixic Acid | - | 10 | 10 | - | 10 | 10 | - | 10 | 10 | - | 10 | 10 |
| | Norfloxacin | - | 10 | 10 | - | 10 | 10 | - | 10 | 10 | - | 10 | 10 |
| Macrolides | 3-O-Acetyltylosin | - | 10 | 10 | 50 | 63 | 80 | - | 10 | 10 | - | 10 | 10 |
| | Erytromycin A | 200 | 228 | 260 | 200 | 228 | 260 | 200 | 228 | 260 | 200 | 228 | 260 |
| | Gamithromycin | - | 10 | 10 | 100 | 112 | 125 | - | 10 | 10 | 50 | 56 | 62 |
| | Neospiramycin | 200 | 252 | 319 | 250 | 316 | 398 | 200 | 252 | 319 | - | 10 | 10 |
| | Spiramycin | 200 | 256 | 327 | 250 | 320 | 409 | 200 | 256 | 327 | - | 10 | 10 |
| | Tildipirosin | 400 | 466 | 542 | 1200 | 1397 | 1626 | - | 10 | 10 | 400 | 466 | 542 |
| | Tilmicosin | 50 | 58 | 68 | 50 | 58 | 68 | 75 | 87 | 102 | 50 | 58 | 68 |
| | Tylosin A | 100 | 125 | 155 | 100 | 125 | 155 | 100 | 125 | 155 | 100 | 125 | 155 |
| | Tylvalosin | - | 10 | 10 | 50 | 66 | 86 | - | 10 | 10 | - | 10 | 10 |
| | Tulathromycin | 300 | 379 | 478 | 800 | 1010 | 1275 | - | 10 | 10 | 450 | 568 | 717 |
| | Tulathromycin Marker | 300 | 342 | 391 | 800 | 913 | 1042 | - | 10 | 10 | 450 | 513 | 586 |
| Sulfonamides | Sulfaquinoxaline | 100 | 113 | 127 | 100 | 113 | 127 | 100 | 113 | 127 | 100 | 113 | 127 |
| | Sulfadiazine | 100 | 113 | 129 | 100 | 113 | 129 | 100 | 113 | 129 | 100 | 113 | 129 |
| | Sulfadimethoxine | 100 | 113 | 128 | 100 | 113 | 128 | 100 | 113 | 128 | 100 | 113 | 128 |
| | Sulfaguanidine | 100 | 116 | 135 | 100 | 116 | 135 | 100 | 116 | 135 | 100 | 116 | 135 |
| | Sulfamerazine | 100 | 110 | 120 | 100 | 110 | 120 | 100 | 110 | 120 | 100 | 110 | 120 |
| | Sulfamethazine | 100 | 112 | 125 | 100 | 112 | 125 | 100 | 112 | 125 | 100 | 112 | 125 |
| | Sulfamethoxazole | 100 | 116 | 134 | 100 | 116 | 134 | 100 | 116 | 134 | 100 | 116 | 134 |
| | Sulfamonomethoxine | 100 | 112 | 126 | 100 | 112 | 126 | 100 | 112 | 126 | 100 | 112 | 126 |
| | Sulfanilamide | 100 | 115 | 131 | 100 | 115 | 131 | 100 | 115 | 131 | 100 | 115 | 131 |
| | Sulfapyridine | 100 | 112 | 125 | 100 | 112 | 125 | 100 | 112 | 125 | 100 | 112 | 125 |
| | Sulfathiazole | 100 | 113 | 128 | 100 | 113 | 128 | 100 | 113 | 128 | 100 | 113 | 128 |
| Diamino pymidines | Trimethoprim | 50 | 56 | 64 | 50 | 56 | 64 | 50 | 56 | 64 | 50 | 56 | 64 |
| Tetracyclines | Chlortetracycline | 100 | 113 | 127 | 100 | 113 | 127 | 100 | 113 | 127 | 100 | 113 | 127 |
| | Doxycycline | 100 | 116 | 134 | 100 | 116 | 134 | 100 | 116 | 134 | 100 | 116 | 134 |
| | Epi-chlortetracycline | 100 | 121 | 147 | 100 | 121 | 147 | 100 | 121 | 147 | 100 | 121 | 147 |
| | Epi-oxytetracycline | 100 | 121 | 147 | 100 | 121 | 147 | 100 | 121 | 147 | 100 | 121 | 147 |

| | | | | | | | | | | | | | |
|---|---|---|---|---|---|---|---|---|---|---|---|---|---|
| | Epi-tetracycline | 100 | 115 | 132 | 100 | 115 | 132 | 100 | 115 | 132 | 100 | 115 | 132 |
| | Oxytetracycline | 100 | 113 | 128 | 100 | 113 | 128 | 100 | 113 | 128 | 100 | 113 | 128 |
| | Tetracycline | 100 | 115 | 133 | 100 | 115 | 133 | 100 | 115 | 133 | 100 | 115 | 133 |
| Lincosamides | Lincomycin | 100 | 112 | 125 | 100 | 112 | 125 | 100 | 112 | 125 | 100 | 112 | 125 |
| Rifampicins | Rifaximin | - | 10 | 10 | - | 10 | 10 | - | 10 | 10 | - | 10 | 10 |
| Pleuromutilins | Tiamulin | - | 10 | 10 | 100 | 121 | 147 | 100 | 121 | 147 | - | 10 | 10 |
| | Valnemulin | - | 10 | 10 | 50 | Not applicable | | - | 10 | 10 | - | 10 | 10 |

**Table S6 –MRL (µg kg$^{-1}$), decision limits (µg kg$^{-1}$) and detection capabilities (µg kg$^{-1}$) of the 64 antibiotics in aquaculture, rabbits, horses and milk**

| Class | Analyte | Aquaculture | | | Rabbit | | | *Equidae* | | | Milk | | |
|---|---|---|---|---|---|---|---|---|---|---|---|---|---|
| | | MRL | CCα | CCβ | MRL | CCα | CCβ | MRL | CCα | CCβ | MRL | CCα | CCβ |
| Amphenicols | Florfenicolamine | 1000 | 1100 | 1210 | 100 | 110 | 121 | 100 | 110 | 121 | - | 10 | 10 |
| | Florfenicol | 1000 | 1262 | 1594 | 100 | 126 | 159 | 100 | 126 | 159 | - | 10 | 10 |
| | Thiamphenicol | 50 | 59 | 70 | 50 | 59 | 70 | 50 | 59 | 70 | 50 | 61 | 74 |
| Beta-lactams | Amoxicillin | 50 | 55 | 61 | 50 | 55 | 61 | 50 | 55 | 61 | 4 | 4.4 | 4.8 |
| | Ampicillin | 50 | 57 | 64 | 50 | 57 | 64 | 50 | 57 | 64 | 4 | 4.7 | 5.4 |
| | Cefacetrile | - | 33 | 33 | - | 33 | 33 | - | 33 | 33 | 125 | Not applicable | |
| | Cefalexin | 200 | 228 | 259 | - | 10 | 10 | - | 10 | 10 | 100 | 111 | 123 |
| | Cefalonium | - | 10 | 10 | - | 10 | 10 | - | 10 | 10 | 20 | 23 | 26 |
| | Cephapirin | - | 10 | 10 | - | 10 | 10 | - | 10 | 10 | 60 | 72 | 86 |
| | Cefazolin | - | 10 | 10 | - | 10 | 10 | - | 10 | 10 | 50 | 61 | 76 |
| | Cefoperazone | - | 10 | 10 | - | 10 | 10 | - | 10 | 10 | 50 | 65 | 84 |
| | Cefquinome | - | 10 | 10 | - | 10 | 10 | 50 | 56 | 63 | 20 | 26 | 34 |
| | Ceftiofur | - | 10 | 10 | 1000 | 1159 | 1343 | 1000 | 1159 | 1343 | 100 | 112 | 125 |
| | Cloxacillin | 300 | 354 | 418 | 300 | 354 | 418 | 300 | 354 | 418 | 30 | 36 | 43 |
| | Desacetylcephapirin | - | 10 | 10 | - | 10 | 10 | - | 10 | 10 | 60 | 66 | 72 |
| | Dicloxacillin | 300 | 349 | 406 | 300 | 349 | 406 | 300 | 349 | 406 | 30 | 36 | 44 |
| | Nafcillin | - | 10 | 10 | - | 10 | 10 | - | 10 | 10 | 30 | 33 | 36 |
| | Oxacillin | 300 | 354 | 418 | 300 | 354 | 418 | 300 | 354 | 418 | 30 | 34 | 39 |
| | Penicillin G | 50 | 57 | 66 | 50 | 57 | 66 | 50 | 57 | 66 | 4 | Not applicable | |
| | Penicillin V | - | 10 | 10 | - | 10 | 10 | - | 10 | 10 | - | 10 | 10 |
| Quinolones | Oxolinic Acid | 100 | 111 | 123 | 100 | 111 | 123 | 100 | 111 | 123 | - | 10 | 10 |
| | Ciprofloxacin | 100 | 114 | 129 | 100 | 114 | 129 | 100 | 114 | 129 | 100 | 112 | 126 |
| | Danofloxacin | 100 | 112 | 125 | 100 | 112 | 125 | 100 | 112 | 125 | 30 | 32 | 35 |
| | Difloxacin | 300 | 336 | 377 | 300 | 336 | 377 | 300 | 336 | 377 | - | 10 | 10 |
| | Enrofloxacin | 100 | 111 | 123 | 100 | 111 | 123 | 100 | 111 | 123 | 100 | 110 | 120 |
| | Flumequine | 600 | 672 | 752 | 200 | 224 | 251 | 200 | 224 | 251 | 50 | 54 | 58 |

| | | | | | | | | | | | | | |
|---|---|---|---|---|---|---|---|---|---|---|---|---|---|
| | Marbofloxacin | - | 10 | 10 | - | 10 | 10 | - | 10 | 10 | 75 | 81 | 88 |
| | Sarafloxacin | 30 | 34 | 38 | - | 10 | 10 | - | 10 | 10 | - | 10 | 10 |
| | Nalidixic Acid | - | 10 | 10 | - | 10 | 10 | - | 10 | 10 | - | 10 | 10 |
| | Norfloxacin | - | 10 | 10 | - | 10 | 10 | - | 10 | 10 | - | 10 | 10 |
| Macrolides | 3-O-Acetyltylosin | - | 10 | 10 | - | 10 | 10 | - | 10 | 10 | - | 10 | 10 |
| | Erytromycin A | 200 | 228 | 260 | 200 | 228 | 260 | 200 | 228 | 260 | 40 | 48 | 57 |
| | Gamithromycin | - | 10 | 10 | - | 10 | 10 | - | 10 | 10 | - | 10 | 10 |
| | Neospiramycin | - | 10 | 10 | - | 10 | 10 | - | 10 | 10 | 200 | 239 | 286 |
| | Spiramycin | - | 10 | 10 | - | 10 | 10 | - | 10 | 10 | 200 | 225 | 253 |
| | Tildipirosin | - | 10 | 10 | - | 10 | 10 | - | 10 | 10 | - | 10 | 10 |
| | Tilmicosin | 50 | 58 | 68 | 50 | 58 | 68 | 50 | 58 | 68 | 50 | 54 | 57 |
| | Tylosin A | 100 | 125 | 155 | 100 | 125 | 155 | 100 | 125 | 155 | 50 | 54 | 58 |
| | Tylvalosin | - | 10 | 10 | - | 10 | 10 | - | 10 | 10 | - | 10 | 10 |
| | Tulathromycin | - | 10 | 10 | - | 10 | 10 | - | 10 | 10 | - | 10 | 10 |
| | Tulathromycin Marker | - | 10 | 10 | - | 10 | 10 | - | 10 | 10 | - | 10 | 10 |
| Sulfonamides | Sulfaquinoxaline | 100 | 113 | 127 | 100 | 113 | 127 | 100 | 113 | 127 | 100 | 108 | 116 |
| | Sulfadiazine | 100 | 113 | 129 | 100 | 113 | 129 | 100 | 113 | 129 | 100 | 114 | 130 |
| | Sulfadimethoxine | 100 | 113 | 128 | 100 | 113 | 128 | 100 | 113 | 128 | 100 | 106 | 113 |
| | Sulfaguanidine | 100 | 116 | 135 | 100 | 116 | 135 | 100 | 116 | 135 | 100 | 123 | 151 |
| | Sulfamerazine | 100 | 110 | 120 | 100 | 110 | 120 | 100 | 110 | 120 | 100 | 114 | 131 |
| | Sulfamethazine | 100 | 112 | 125 | 100 | 112 | 125 | 100 | 112 | 125 | 100 | 123 | 151 |
| | Sulfamethoxazole | 100 | 116 | 134 | 100 | 116 | 134 | 100 | 116 | 134 | 100 | 111 | 123 |
| | Sulfamonomethoxine | 100 | 112 | 126 | 100 | 112 | 126 | 100 | 112 | 126 | 100 | 116 | 135 |
| | Sulfanilamide | 100 | 115 | 131 | 100 | 115 | 131 | 100 | 115 | 131 | 100 | 120 | 143 |
| | Sulfapyridine | 100 | 112 | 125 | 100 | 112 | 125 | 100 | 112 | 125 | 100 | 116 | 134 |
| | Sulfathiazole | 100 | 113 | 128 | 100 | 113 | 128 | 100 | 113 | 128 | 100 | 114 | 129 |
| Diamino pymidines | Trimethoprim | 50 | 56 | 64 | 50 | 56 | 64 | 100 | 113 | 127 | 50 | 54 | 58 |
| Tetracyclines | Chlortetracycline | 100 | 113 | 127 | 100 | 113 | 127 | 100 | 113 | 127 | 100 | 112 | 125 |
| | Doxycycline | 100 | 116 | 134 | 100 | 116 | 134 | 100 | 116 | 134 | - | 10 | 10 |
| | Epichlortetracycline | 100 | 121 | 147 | 100 | 121 | 147 | 100 | 121 | 147 | 100 | 118 | 139 |
| | Epioxytetracycline | 100 | 121 | 147 | 100 | 121 | 147 | 100 | 121 | 147 | 100 | 125 | 155 |

| | | | | | | | | | | | | | |
|---|---|---|---|---|---|---|---|---|---|---|---|---|---|
| | Epitetracycline | 100 | 115 | 132 | 100 | 115 | 132 | 100 | 115 | 132 | 100 | 112 | 126 |
| | Oxytetracycline | 100 | 113 | 128 | 100 | 113 | 128 | 100 | 113 | 128 | 100 | 109 | 118 |
| | Tetracycline | 100 | 115 | 133 | 100 | 115 | 133 | 100 | 115 | 133 | 100 | 108 | 117 |
| Lincosamides | Lincomycin | 100 | 112 | 125 | 100 | 112 | 125 | 100 | 112 | 125 | 150 | 161 | 172 |
| Rifampicins | Rifaximin | - | 10 | 10 | - | 10 | 10 | - | 10 | 10 | 60 | 69 | 80 |
| Pleuromutilins | Tiamulin | - | 10 | 10 | 100 | 121 | 147 | - | 10 | 10 | - | 10 | 10 |
| | Valnemulin | - | 10 | 10 | 50 | Not applicable | | - | 10 | 10 | - | 10 | 10 |

**Table S7 – Estimated LODs and LOQs based on the observed accuracy at the first and at the second validation level**

| | Muscle | | Milk | |
|---|---|---|---|---|
| **Analyte[a]** | **LOD ($\mu g\ kg^{-1}$)** | **LOQ ($\mu g\ kg^{-1}$)** | **LOD ($\mu g\ kg^{-1}$)** | **LOQ ($\mu g\ kg^{-1}$)** |
| All analytes except the antibiotics listed below | 10 | 10 | 10 | 10 |
| Tulathromycin | 10 | 10 | 10 | 10 |
| Valnemulin | 10 | - | 10 | 10 |
| Cefacetrile | 33 | 33 | 33 | - |
| Tulathromycin marker | 10 | 10 | 10 | - |
| Amoxicillin | 10 | 10 | 2 | 2 |
| Ampicillin | 10 | 10 | 2 | 2 |
| Penicillin G | 10 | 10 | 2 | 10 |

[a]For valnemulin (muscle) and cefacetrile and tulathromycin marker (milk) the method can be used only for screening purposes (inadequate accuracy);

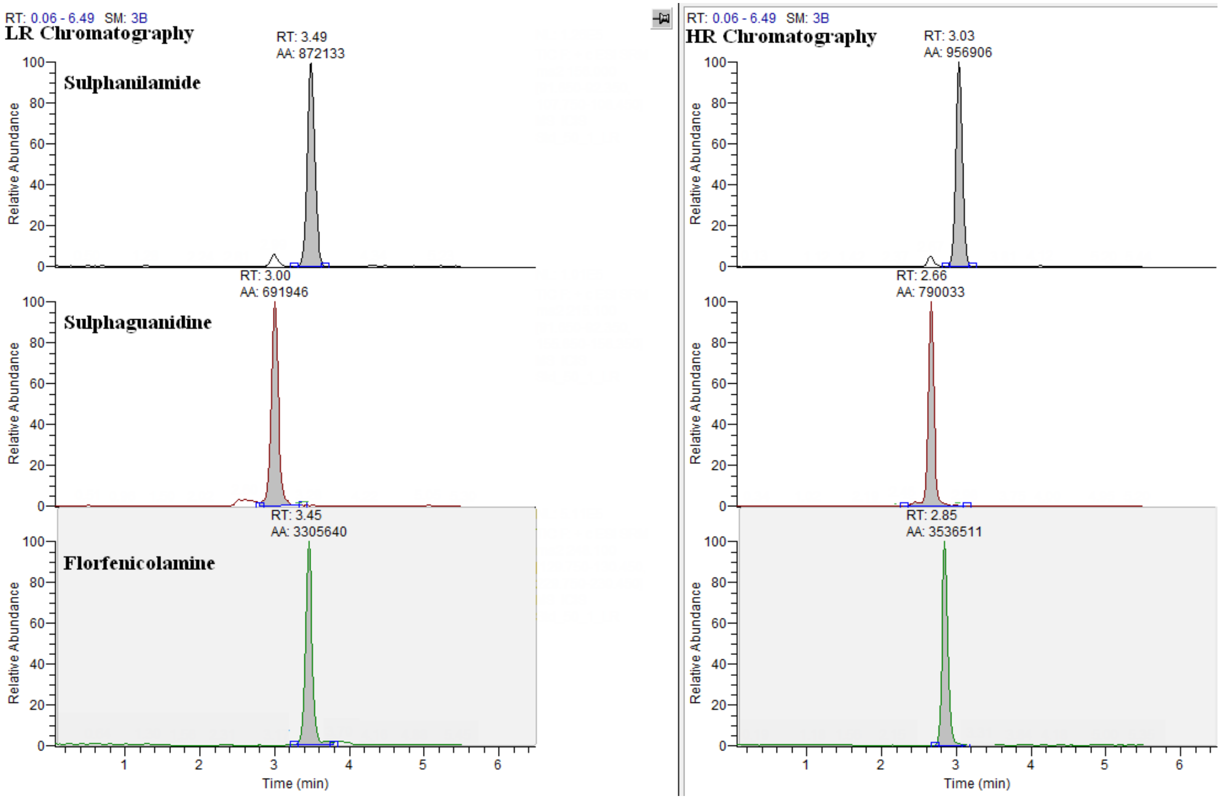


Figure S1- LC-QqQ chromatograms of the three more polar analytes (sulfanilamide, sulfaguanidine and florfenicolamine) with different % MeOH at the beginning of the gradient: 2% (left) and 5% (right)

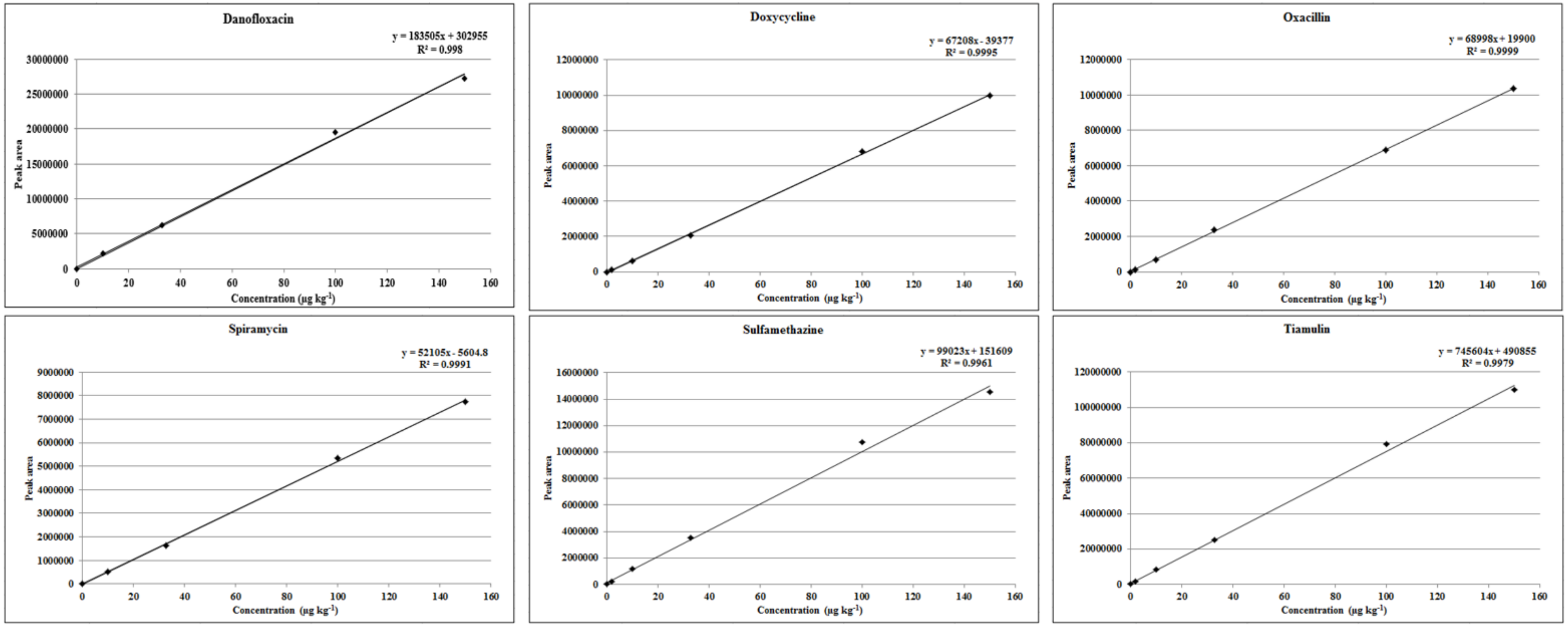


Figure S2 – Matrix-matched calibration curves of danofloxacin, doxycycline, oxacillin, spiramycin, sulfamethazine and tiamulin in meat

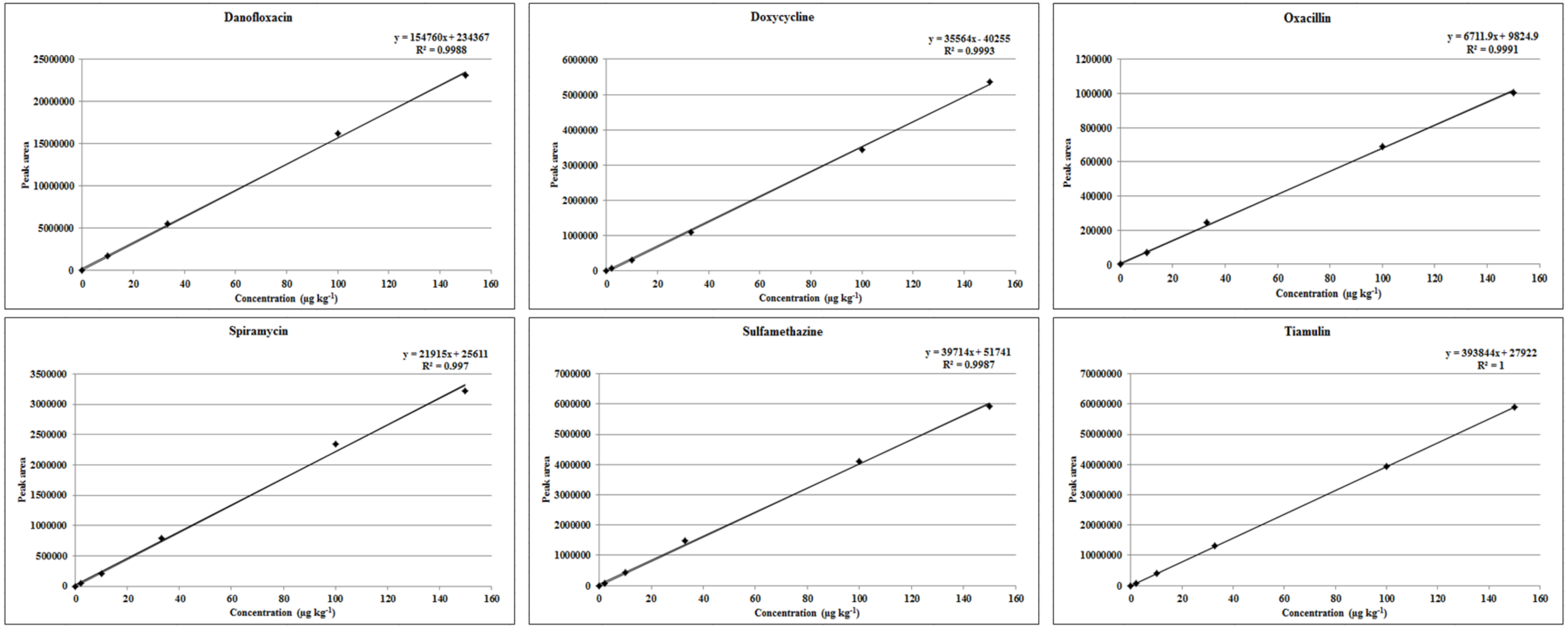


Figure S3 – Matrix-matched calibration curves of of danofloxacin, doxycycline, oxacillin, spiramycin, sulfamethazine and tiamulin in milk

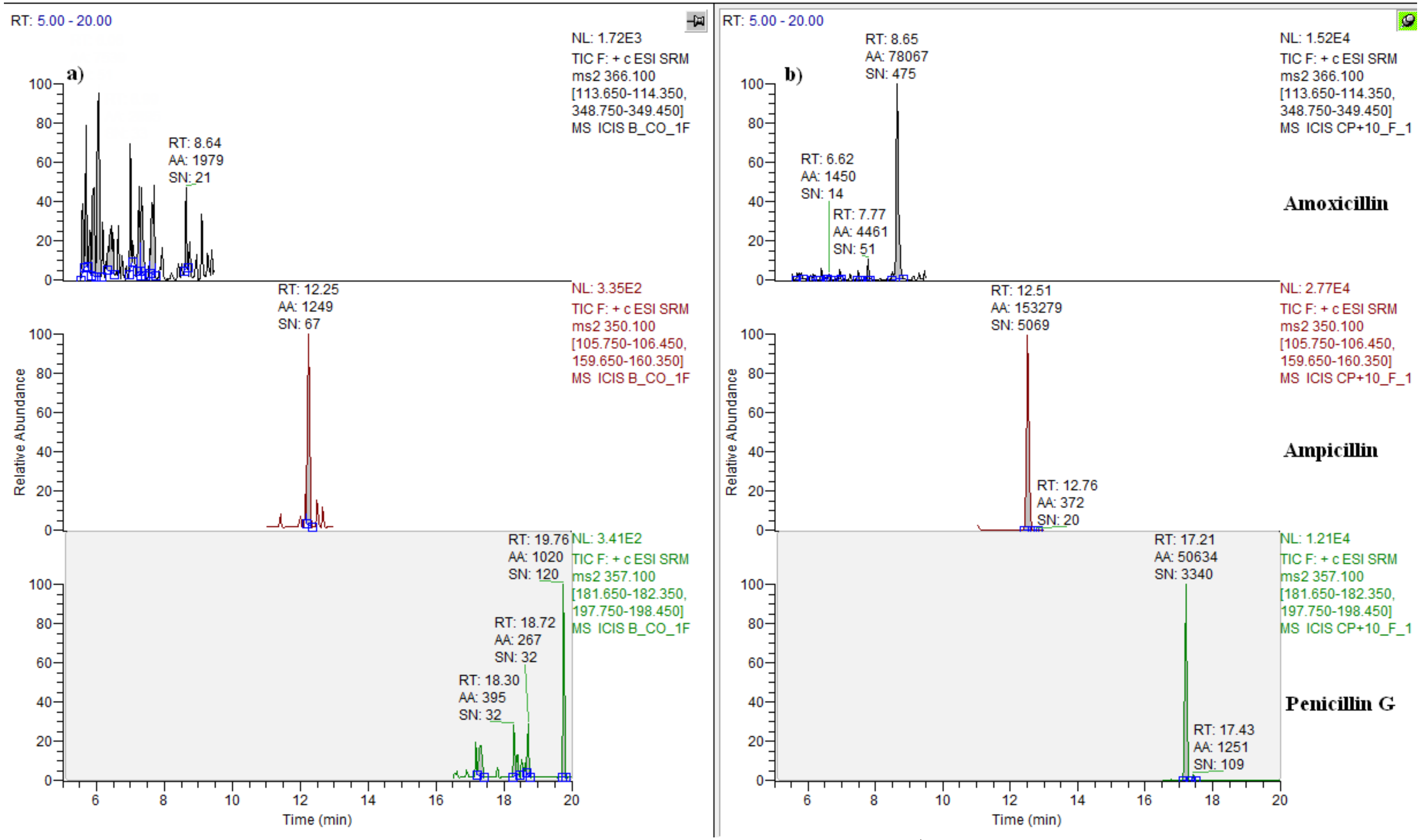


Figure S4- LC-QqQ chromatograms of a blank (a) and a milk sample spiked at 2 μg $kg^{-1}$ (b) of the three penicillins with MRL at 4 μg $kg^{-1}$

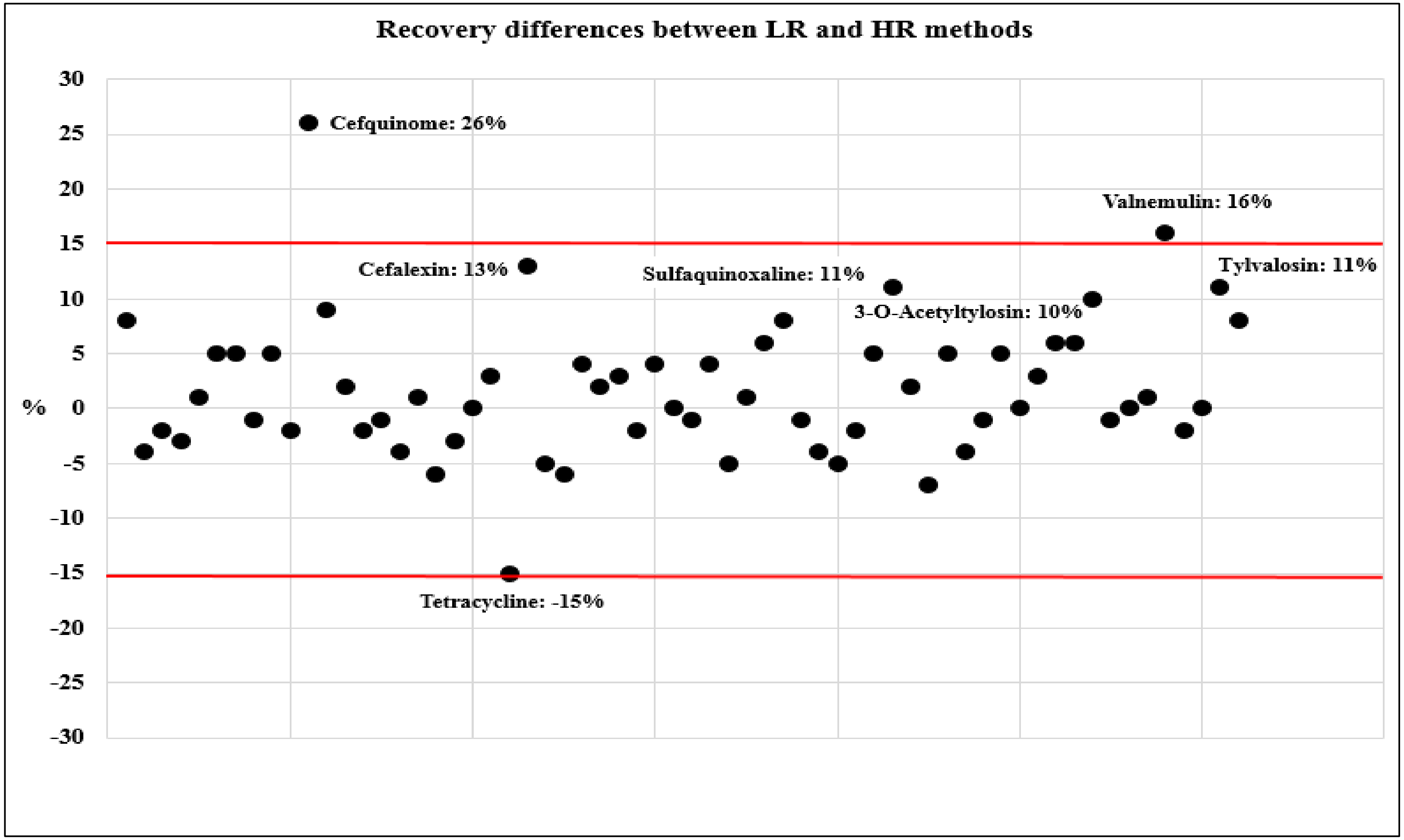


Figure S5 – Recovery differences between the LC-QqQ and LC-Q-Orbitrap methods in meat. Positive values (%) indicate better recovery of LC-QqQ procedure and vice versa

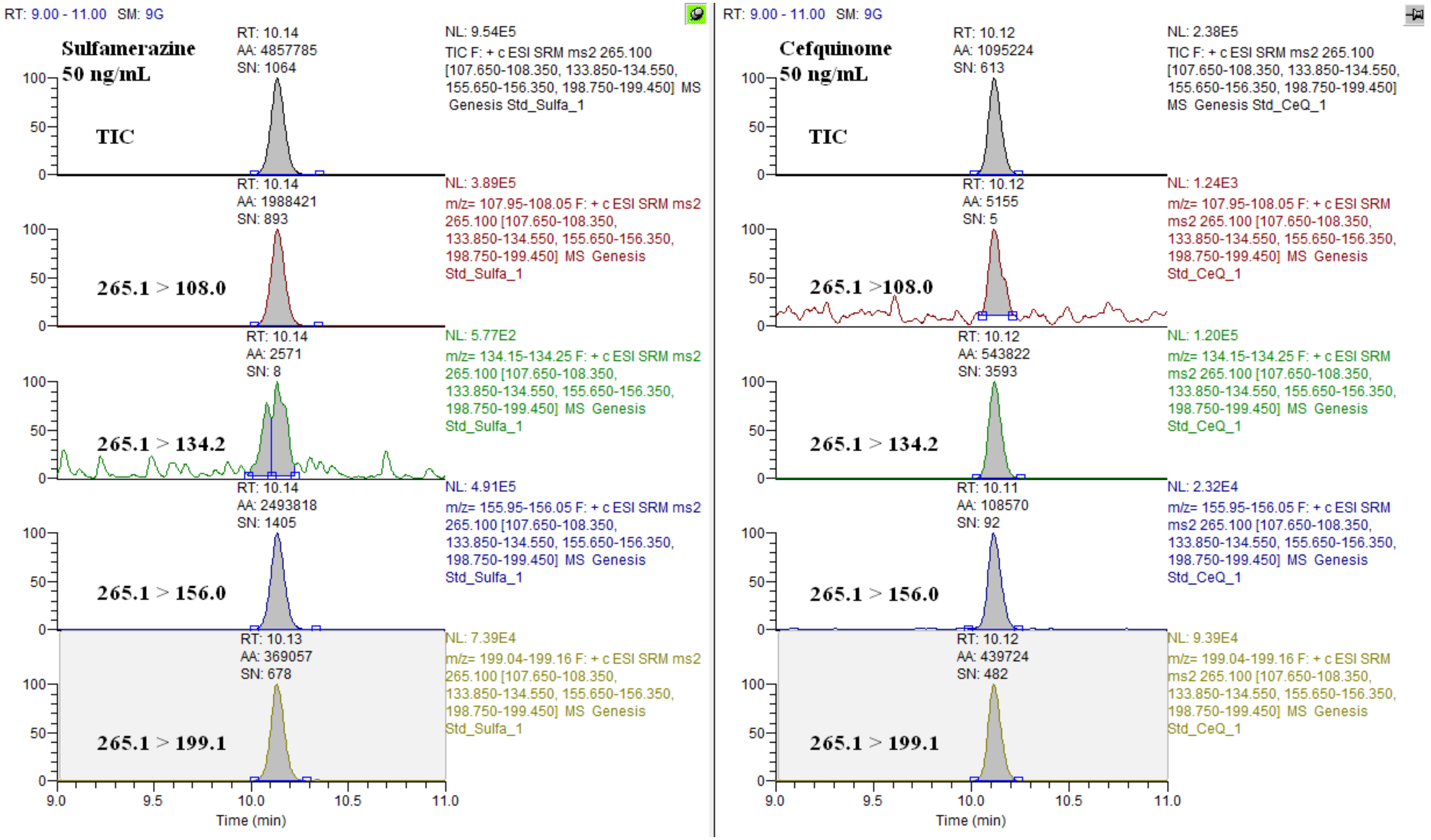


Figure S6 – LC-QqQ chromatograms of individual solutions (50 ng $mL^{-1}$) of sulfamerazine (left) and cefquinome (right)

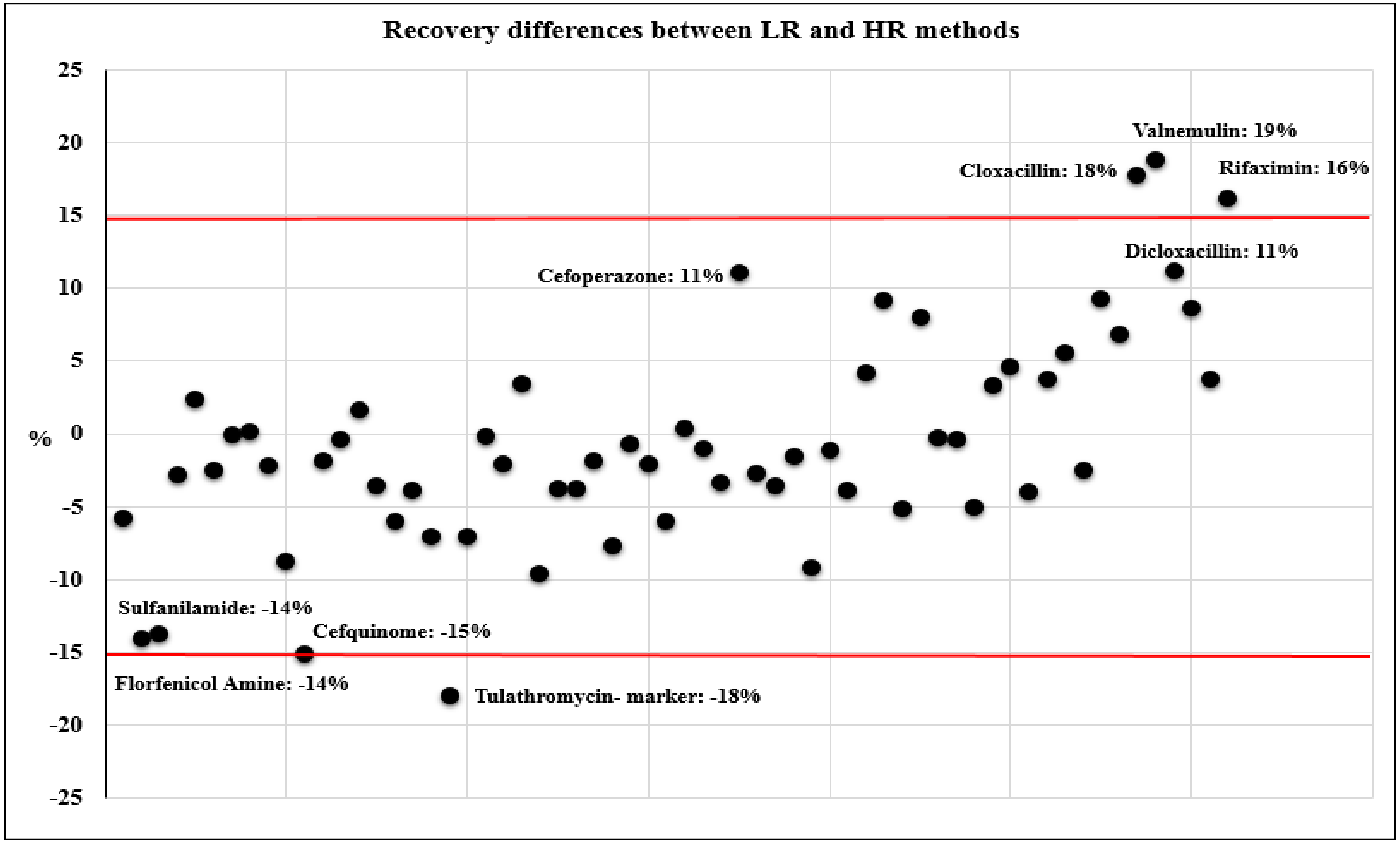


Figure S7 – Recovery differences between the LC-QqQ and LC-Q-Orbitrap methods in bovine milk. Positive values (%) indicate better recovery of LC-QqQ procedure and vice versa

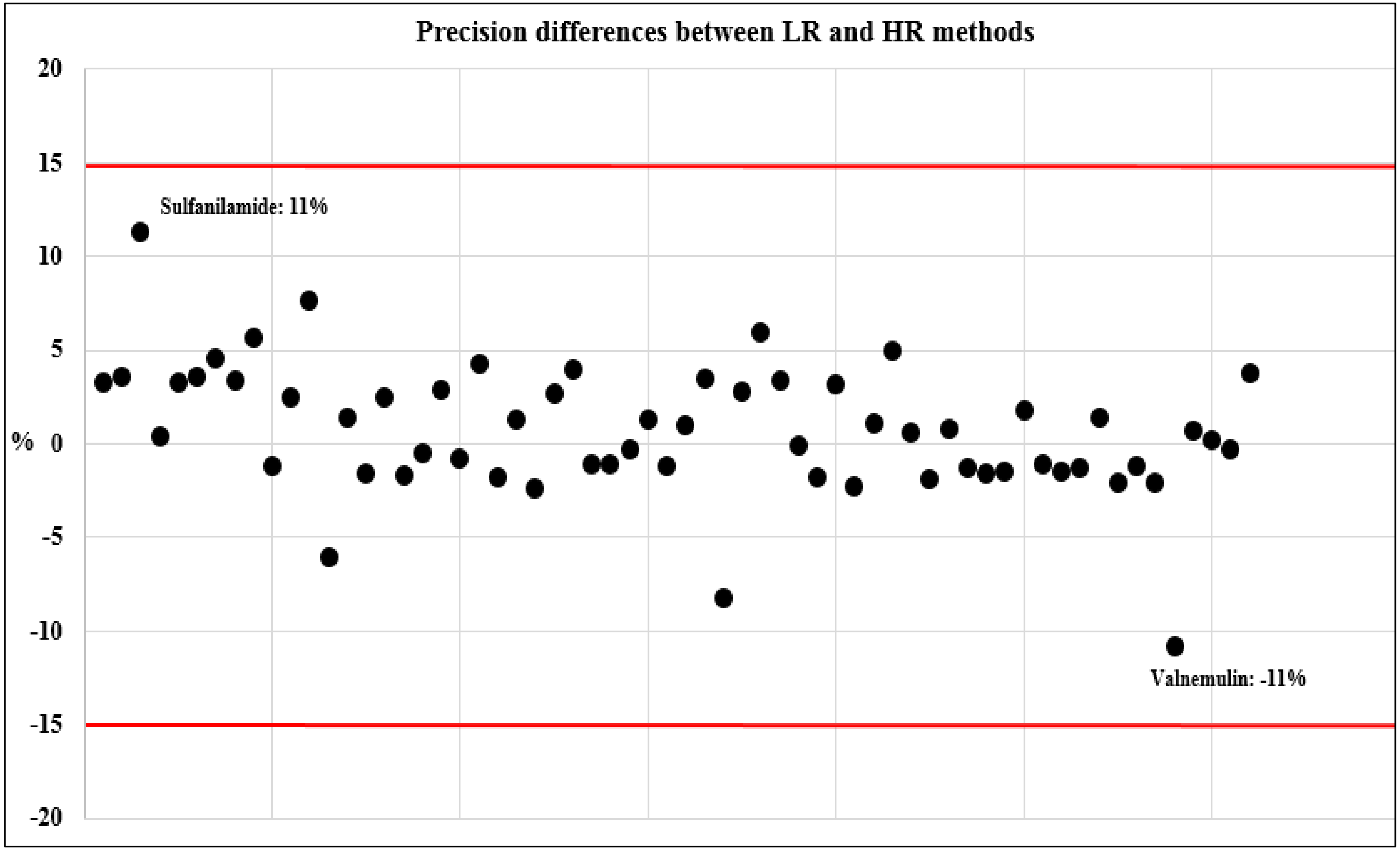

Figure S8 – Precision differences between the LC-QqQ and LC-Q-Orbitrap methods in meat. Positive values (%) indicate better precision of LC-QqQ procedure and vice versa

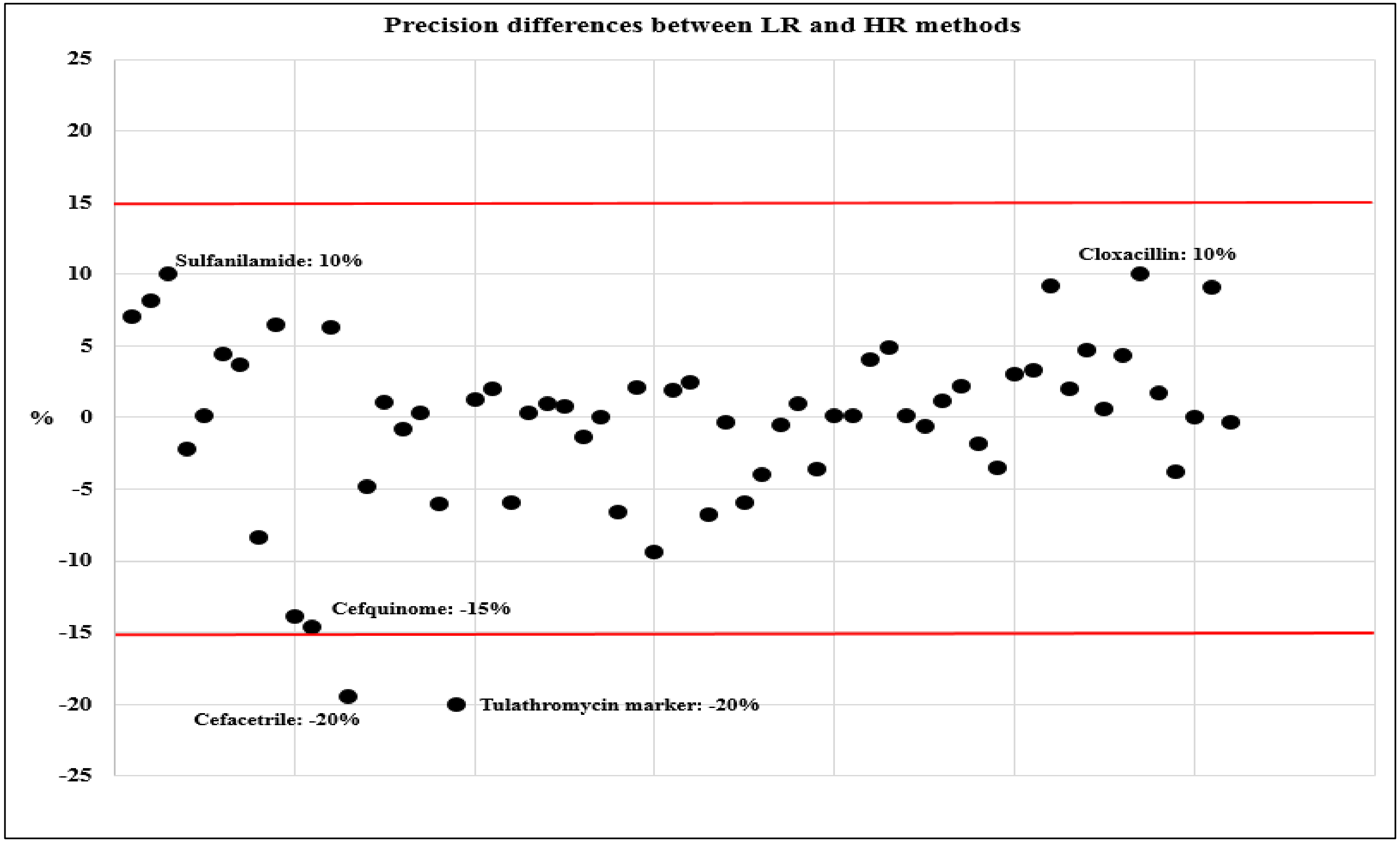


Figure S9 – Precision differences between the LC-QqQ and LC-Q-Orbitrap methods in bovine milk. Positive values (%) indicate better precision of LC-QqQ procedure and vice versa
